# Laparoscopic antireflux surgery increases health-related quality of life in children with GERD

**DOI:** 10.1007/s00464-016-5336-5

**Published:** 2016-11-18

**Authors:** Femke A. Mauritz, Rebecca K. Stellato, L. W. Ernst van Heurn, Peter D. Siersema, Cornelius E. J. Sloots, Roderick H. J. Houwen, David C. van der Zee, Maud Y. A. van Herwaarden-Lindeboom

**Affiliations:** 1 Department of Pediatric Surgery, Wilhelmina Children’s Hospital, University Medical Center Utrecht, Room: KE.04.140.5, PO Box 85090, 3508 AB Utrecht, The Netherlands; 2 Department of Pediatric Gastroenterology, Wilhelmina Children’s Hospital, University Medical Center Utrecht, Utrecht, The Netherlands; 3 Department of Gastroenterology and Hepatology, University Medical Center Utrecht, Utrecht, The Netherlands; 4 Department of Biostatistics, Julius Center for Health Sciences and Primary Care, University Medical Center Utrecht, Utrecht, The Netherlands; 5 Department of Pediatric Surgery, Maastricht University Medical Center, Maastricht, The Netherlands; 6 Department of Pediatric Surgery, Sophia Children’s Hospital, Erasmus MC, Rotterdam, The Netherlands

**Keywords:** Pediatric, Children, Reflux, GERD, Fundoplication, Quality of life, Antireflux surgery

## Abstract

**Introduction:**

Improving health-related quality of life (HRQoL) is increasingly recognized as an essential part of patient care outcome. Little is known about the effect of laparoscopic antireflux surgery (LARS) on the HRQoL in the pediatric patients. The aims of this study were to evaluate the effect of LARS on HRQoL in children with gastroesophageal reflux disease (GERD) and to identify predictors that influence HRQoL outcome after LARS.

**Methods:**

Between 2011 and 2013, 25 patients with therapy-resistant GERD [median age 6 (2–18) years] were included prospectively. Caregivers and children with normal neurodevelopment (>4 years) were asked to fill out the validated PedsQL 4.0 Generic Core Scales before and 3–4 months after LARS.

**Results:**

The PedsQL was completed by all caregivers (*n* = 25) and 12 children. HRQoL total score improved significantly after LARS, both from a parental (*p* = 0.009) and child’s perspective (*p* = 0.018). The psychosocial health summary and physical health summary scores also improved significantly after LARS. HRQoL before and after LARS was significantly lower in children with impaired neurodevelopment (*p* < 0.001). However, neurodevelopment did not influence the effect of LARS on HRQoL. The only significant predictor for improvement in HRQoL after LARS was age at the time of operation (*p* = 0.001).

**Conclusions:**

HRQoL significantly improves after LARS. Although children with impaired neurodevelopment had lower overall HRQoL, neurodevelopment by itself does not predict inferior improvement in HRQoL after LARS. Older children have a more favorable HRQoL outcome after LARS compared to younger children. This may suggest caution when considering LARS in younger GERD patients.

Laparoscopic antireflux surgery (LARS) is an established treatment option performed in pediatric patients with severe gastroesophageal reflux disease (GERD) resistant to medical treatment [1, 2]. LARS primarily aims to decrease (acid) reflux events and to reduce reflux symptoms. However, as shown in earlier studies the effect on reflux symptoms does not always correlate to more objective assessments of success of therapy [3, 4]. Furthermore, comorbidities (e.g., impaired neurodevelopment) and complications, such as dysphagia and gas-bloat syndrome [5], may also affect success of therapy.

To better assess the impact of pediatric diseases and treatments from the perspective of the pediatric patient and their caregivers health-related quality of life (HRQoL), assessment has been increasingly recognized as an essential part of patient care outcome [6]. Effects of LARS on HRQoL have been mainly investigated in adult population. These studies almost all showed that HRQoL improves after LARS [7–9]. In the pediatric population, only few studies have focused on this outcome parameter [10–12]. HRQoL in these studies improves; however, none of these studies have used pediatric validated questionnaires. In two studies [10, 11], a questionnaire designed for adults had been modified for pediatric use and one study had only used parental proxy report to score HRQoL [12]. Furthermore, none of these studies could identify determinants that influence HRQoL outcome after LARS. The Pediatric Quality of Life Inventory (http://www.pedsql.org.) 4.0 Generic Core Scales (PedsQL) is a reliable and valid tool (also for the Dutch language) for parental proxy report and parallel child’s self-report on HRQoL. It has been used to assess HRQoL in children with numerous acute and chronic health conditions, as well as in healthy populations [6, 13–17]. The aim of this study was to evaluate the effect of LARS on HRQoL using the PedsQL and to identify predictors that may influence HRQoL outcome after LARS.

## Patients and methods

We performed a prospective multicenter study in three University Medical Centers in the Netherlands performing laparoscopic fundoplication in children (Wilhelmina children’s Hospital, University Medical Center Utrecht (UMCU): Sophia children’s Hospital, Erasmus University Medical Center (EMC) and Maastricht University Medical Center (MUMC). From July 2011 until December 2013, we prospectively included all pediatric patients diagnosed with PPI-therapy-resistant GERD. Patients that had undergone previous esophageal or gastric surgery (except previous gastrostomy placement) and those who had structural abnormalities other than an esophageal hiatal hernia were excluded.

### Patients

In total, 25 children were included. Mean age of the included patients was six (range 2–18) years at the time of fundoplication (Table 1). Impaired neurodevelopment was present in 20% of patients (5/25 patients). Causes of impaired neurodevelopment are shown in Table 2.

**Table 1:** Baseline characteristics

	Median (IQR)
Age at time of operation (years)	6.0 (3.0–11.0)
Duration of hospital admission (days)	3.0 (2.0–4.5)

	*n* (%)
Male gender	12 (48.0%)
Neurological normal development	20 (80.0%)
Gastrostomy preoperatively in situ	4 (16.0%)

**Table 2:** Neurological impairment (*n* = 5)

Charge syndrome
Mitochondrial complex II deficiency
Post-hypoxic encephalopathy
Congenital rubella infection
Neurologically impairment of unknown origin with autistic behavior

### Surgical procedures

All laparoscopic fundoplications were performed by experienced pediatric surgeons in pediatric laparoscopic surgery. In the UMCU the anterior, partial fundoplication according to Thal [18] was used to perform fundoplication. In the other two UMC’s (EMC and MUMC) the posterior, total fundoplication according to Nissen [19] was performed. Before fundoplication, the distal esophagus was fully mobilized; the distal 3 cm of the esophagus was repositioned back into the abdomen. Both vagal nerves were identified, and after dissection of both crura the hiatus was closed routinely (UMCU and EMC). Thereafter, the fundoplication was constructed. The Thal fundoplication was performed by plicating the fundus of the stomach over 270° against the distal anterior intra-abdominal part of the esophagus and the diaphragmatic crus [3, 18]. A floppy Nissen was constructed with one of the sutures of the 360° posterior wrap incorporated in the esophageal wall [19].

### Clinical assessment

Before and 3 months after laparoscopic fundoplication, clinical assessment was performed using the PedsQL 4.0 Generic Core Scale for HRQoL, a reflux-specific symptom questionnaire, 24-h multichannel intraluminal impedance-pH monitoring (MII-pH monitoring) and an 13C-labeled Na-octanoate breath test. Surgical re-interventions, type and indication for re-intervention, endoscopic procedures, complications, and comorbidities were registered in a prospective database.

1. Health-related Quality of Life—caregivers and children with normal neurodevelopment (>4 years) were asked to fill out the 23-items PedsQL 4.0 Generic Core Scales [1, 14, 17, 19–21]. The scales are available for parental proxy report, subdivided in four age-adjusted questionnaires (ages: 2–4; 5–7; 8–12; and 13–18 years) and as a parallel child’s self-report (ages: 5–7; 8–12; and 13–18 years). The PedsQL 4.0 Generic Core Scales comprises four domains: physical functioning (8 items), emotional functioning (5 items), social functioning (5 items), and school functioning (5 items). With the four domains, the physical health summary score, the psychosocial summary score and the total score are calculated. The physical health summary score is reflected by the physical functioning scale. The psychosocial health summary score is reflected by the mean of the other three domains (emotional, social and school functioning). Scale scores per domain were computed as the sum of the items divided by the number of items answered. Thereafter, items were reverse-scored and transformed to a 0–100 scale. Higher scale scores indicate better HRQoL.
2. Reflux-specific questionnaire—patients and/or their parents were asked to fill out the Gastroesophageal Reflux Symptom Questionnaire [22].
3. Ambulatory 24-h MII-pH monitoring—MII-pH monitoring was performed using an age-adjusted combined impedance-pH catheter (Unisensor AG, Attikon, Switzerland). Pathological acid exposure was defined as total acid exposure time ≥6, ≥9% in upright, and ≥3% in the supine body position [23, 24]. The symptom index (SI) and the symptom association probability (SAP) was calculated when the patients experienced symptoms during measurement [25, 26].
4. Gastric emptying breath test—to assess gastric emptying time, we used a 13C-labeled Na-octanoate breath test [27]. Gastric emptying half time is defined as the time when the first half of the 13C-labeled substrate has been metabolized, that is, when the cumulative excretion of 13C in the breath is half the ingested amount. Gastric emptying percentiles were calculated according to the reference values obtained by van den Driessche et al. [28].

### Ethical approval and trial registration

This study was registered at the start of the study in the Dutch national trial registry (www.trailregister.nl; Identifier: 2934). Ethical approval for this prospective multicenter study was obtained from the University Medical Center Utrecht Ethics Committee, and local approval was obtained by the remaining two participating centers. Prior to any trial-related study procedure, informed consent from the patients’ parents and children (≥12 years) was obtained.

### Statistical analysis

Continuous variables, when symmetric, were expressed as mean ± standard error. Skewed variables were expressed as median with interquartile ranges (IQR). For statistical analysis, we used the paired sample *t* test or the Wilcoxon signed-ranks test. The McNemar–Bowker test was used to compare groups in case of nominal outcome measures. To assess the relationship between HRQoL and age at the time of operation, impaired neurodevelopment, reflux symptoms, acid exposure and gastric emptying, we used a linear mixed model with a random intercept per patient. A mixed model allowed us to analyze preoperative and postoperative measurements simultaneously, while taking into account correlation of measurements from the same subjects. Backwards selection was performed using the AIC. A linear regression analysis was performed to identify determinants influencing HRQoL and the effect of LARS on HRQoL. Determinant of interest included: age at the time of operation, impaired neurodevelopment, reflux symptoms, preoperative acid exposure time and preoperative gastric emptying rate. Differences with a *p* < 0.05 were considered statistically significant. All analyses were performed using IBM^®^ 22.0.0 SPSS statistical package (IBM, Armonk, NY).

## Results

In total, 18 Thal and 7 Nissen fundoplications were performed. In all patients, fundoplication was completed by laparoscopy. Perioperative complications were not observed. One patient with retching due to impaired neurodevelopment developed severe recurrent reflux caused by hiatal herniation that required re-fundoplication. In six children, temporary nasogastric tube feedings were required to obtain sufficient caloric intake. This was caused by transient dysphagia (dysphagia dissolved within 3–4 months after LARS; *n* = 4), persistent dysphagia (>3–4 months after LARS; *n* = 1) or refusal of oral feedings (*n* = 1).

### Health-related quality of life

**Fig. 1:**
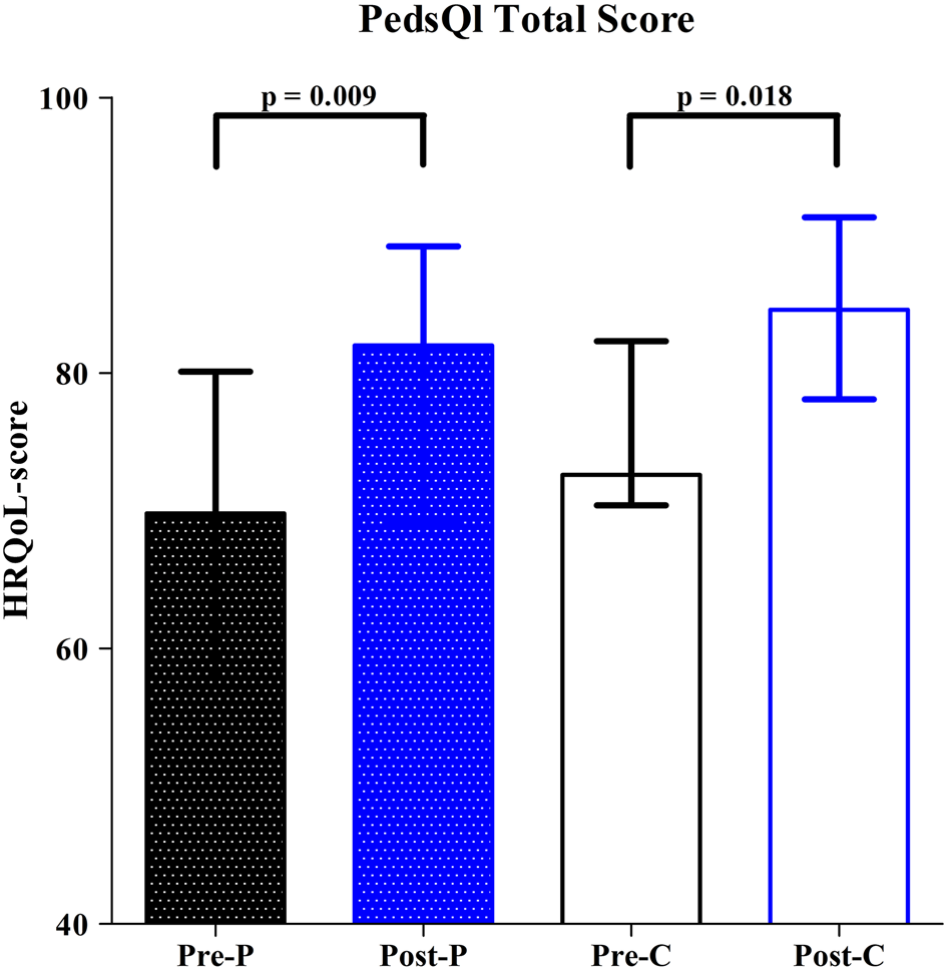
HRQoL assessment using the PedsQL—total score (pre = before LARS; post = after LARS; *P* = parental proxy report; C = child’s self-report)

The PedsQL was completed by all caregivers both before and after LARS for all included patients (*n* = 25). The HRQoL total score improved significantly after LARS from 69.8 (57.2–80.1) to 82.0 (69.3–89.2; *p* = 0.009; Fig. 1a). Twelve children were able to fill out the parallel self-report, and their total score also improved significantly from 72.6 (7.4–82.3) to 84.6 (78.1–91.3; *p* = 0.018; Fig. 1).

**Fig. 2:**
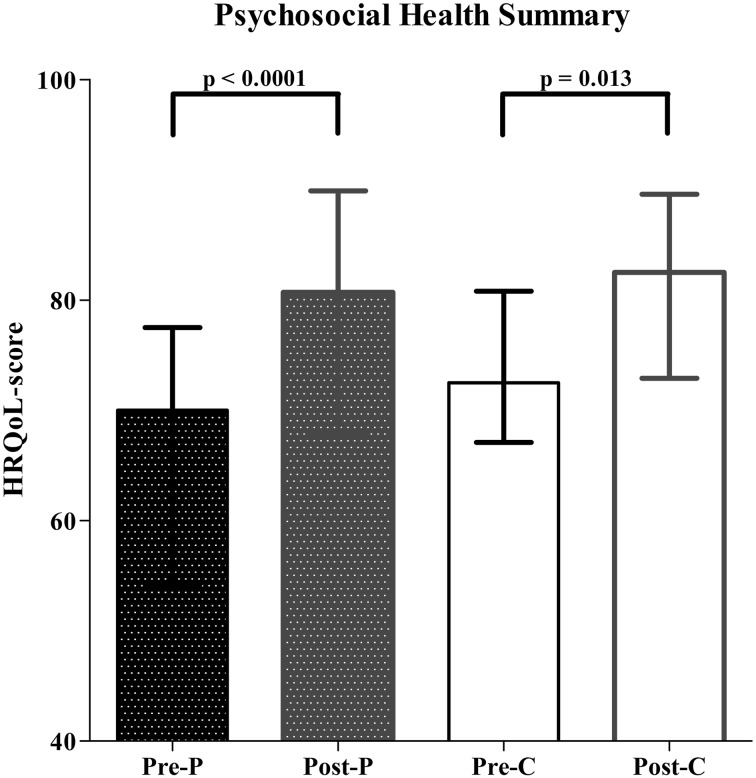
HRQoL assessment using the PedsQ—psychosocial health summary (pre = before LARS; post = after LARS; *P* = parental proxy report; C = child’s self-report)

**Fig. 3:**
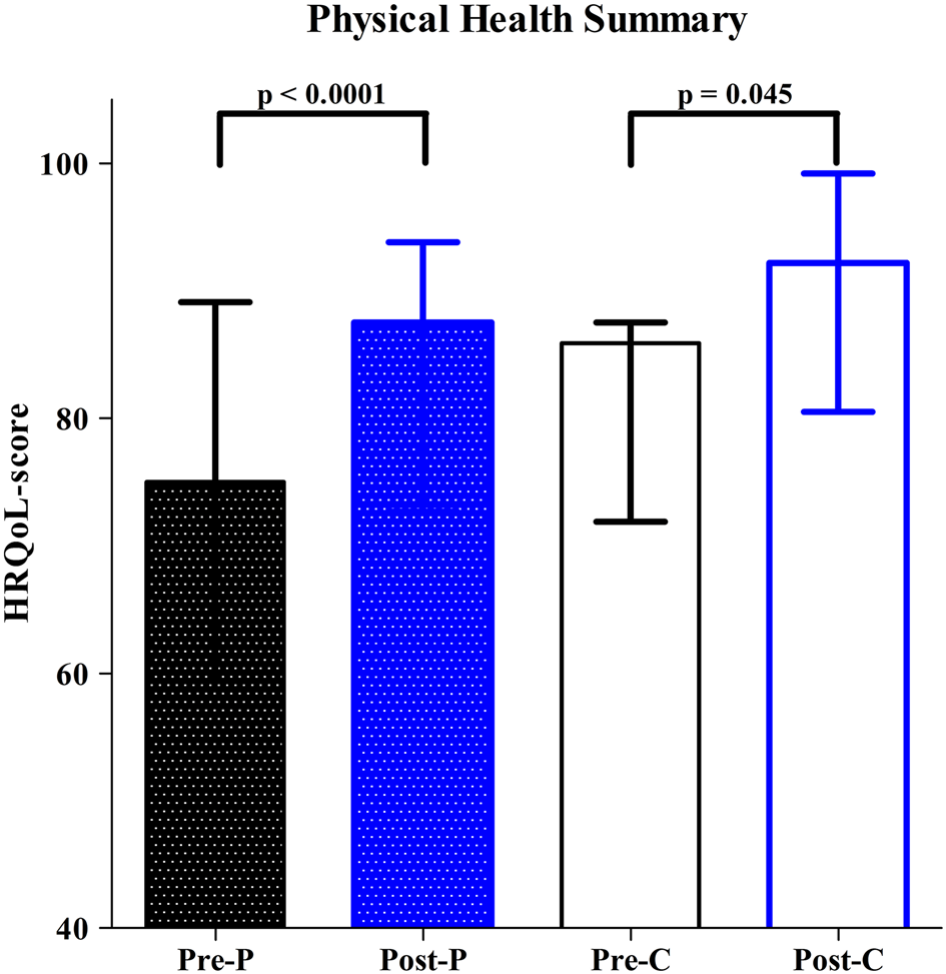
HRQoL assessment using the PedsQL—physical health summary (pre = before LARS; post = after LARS; *P* = parental proxy report; C = child’s self-report)

Furthermore, the psychosocial [54.2 (69.7–77.5) to 82.5 (72.9–89.6); *p* < 0.0001] and the physical health summary [75.0 (59.4–89.1) to 92.2 (80.5–99.2); *p* < 0.0001] also significantly improved for both caregivers as well as children’s self-report after LARS (Figs. 2, 3).

Patients’ self-report of overall HRQoL outcomes was significantly higher (*p* = 0.037) than parental proxy report before LARS; self-reported and proxy-reported HRQoL scores after LARS were not different.

### Reflux symptoms

Reflux symptoms significantly decreased from 16 (64%) patients with severe reflux symptoms before LARS to one (4%) patient after LARS (*p* = 0.001). Deterioration of symptom severity or frequency was not seen in any of the patients. Dysphagia was reported in seven (28%) patients before and in eight (32%) patients after LARS (*p* = 0.887). New-onset dysphagia was seen in three of these eight patients after LARS (Table 3).

**Table 3:** Symptoms (*n* = 25)

	Preoperative (*n*; %)	3–4 months postoperative (*n*; %)	*p* value
Reflux symptoms			
No symptoms	0 (0%)	17 (68%)	0.001
Mild reflux symptoms	2 (8%)	5 (20%)	
Moderate reflux symptom*s*	7 (28%)	2 (8%)	
Severe reflux symptoms	16 (64%)	1 (4%)	
Dysphagia	7 (28%)	8 (32%)	0.887

Gastroesophageal functional assessment tests—total acid exposure decreased significantly from 8.5% (IQR 2.5–32.8) to 0.8% (IQR 0–21.6) after LARS (*p* < 0.0001). Median gastric emptying rate before LARS (percentile 75, IQR 3–99) was similar to that after LARS (70, IQR 5–99, *p* = 0.530).

### Factors influencing HRQoL

Patients with impaired neurodevelopment (NI) had significantly lower HRQoL compared to patients with normal neurodevelopment (NN) (estimate 23.4; *p* = 0.006; (95% CI 7.2–39.5). Furthermore, reflux symptoms were also negatively associated with lower HRQoL (estimate = −4.3 l; *p* = 0.006; 95% CI −7.5 to −1.3). Age at the time of operation (*p* = 0.11), gastric emptying (*p* = 0.82) and total acid exposure (*p* = 0.75) did not significantly influence HRQoL.

### Predictors for the effect of LARS on HRQoL

**Fig. 4:**
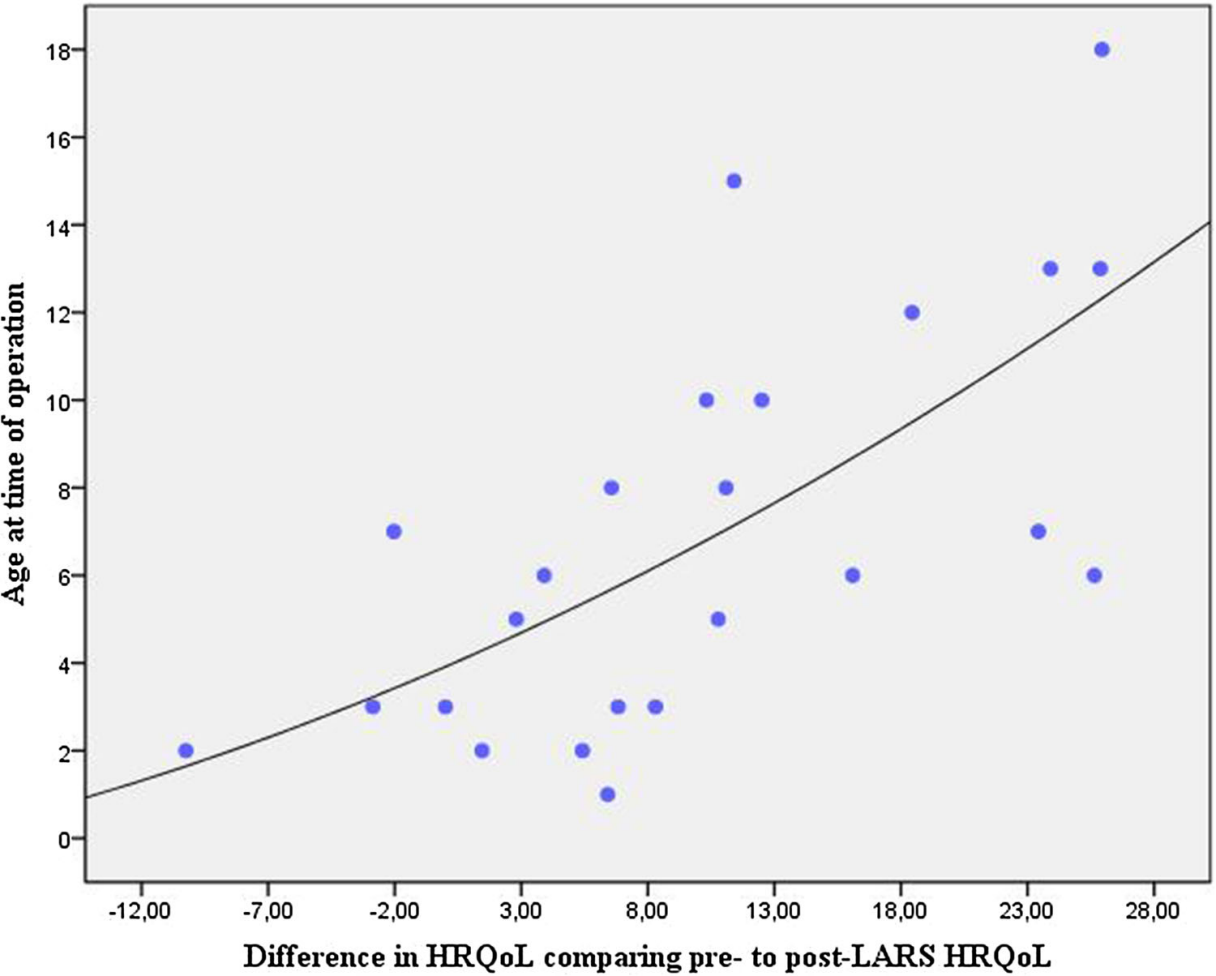
Scatterplot illustrating difference in HRQoL comparing pre- to post-LARS HRQoL

Linear regression analysis showed that an increase in age at the time of operation was a significant predictor for improvement in HRQoL after LARS (*p* = 0.001; estimate = 1.6; 95% CI 0.8–2.5; Fig. 4). Although HRQoL was significantly lower in NI children, neurodevelopment itself did not influence the change in HRQoL (*p* = 0.73). Preoperative gastric emptying rate, total acid exposure time and reflux symptoms also did not significantly influence the change in HRQoL (Table 4).

**Table 4:** Predictors for the effect of LARS on HRQoL

	Estimate	*p* value	95% CI
Age at time of operation	1.7	0.001	0.8–2.5
Neurological development	1.8	0.73	−9.3–12.9
Preoperative reflux symptoms	2.8	0.61	−8.7–14.4
Preoperative acid exposure time (%)	0.3	0.28	−0.3–0.9
Preoperative gastric emptying	−0.004	0.94	−0.1–0.1

Linear regression analysis (95% CI, 95% confidence interval)

## Discussion

This is the first study on HRQoL in children undergoing LARS that used a validated pediatric HRQoL questionnaire [6, 13–17]. We demonstrate that after LARS HRQoL significantly increases and after LARS HRQoL scored were comparable to the normal HRQoL scores measured in a healthy population [13]. Furthermore, age at the time of operation is a significant predictor for improvement in HRQoL after LARS. Previous studies also showed a significant increase in HRQoL [10–12]. In two studies [10, 11], a questionnaire designed for adults had been modified for pediatric use and the third study only used parental proxy report. In contrast to these previous studies, this is the first study in pediatric LARS using a validated questionnaire for HRQoL. Using validated questionnaires in the pediatric population is important as results and questionnaires are not simply translatable for pediatric uses because pathophysiology, and patterns and symptoms of diseases may be different in children compared to adults [29]. Furthermore, this is the first study using both parental proxy reports as well as child’s self-reports.

Some authors hypothesize that children with impaired neurodevelopment (NI) may not benefit to the same extent from LARS as those with normal development [30–32]. In the current study, overall HRQoL was significantly lower in NI children. However, the neurodevelopment itself did not influence the change in HRQoL. This indicates that while NI children with GERD had a lower overall HRQoL, LARS was equally effective regarding the change in HRQoL compared to children with normal neurodevelopment (NN). It is not surprising that NI children, who have more (co-) morbidity than NN children, scored lower in HRQoL; the PedsQL is able to distinguish between healthy children and pediatric patients with acute or chronic health conditions, and it is related to indicators of morbidity and illness burden [21].

The HRQoL scores of NI children for physical, social and school functioning were scored significantly lower compared to NN children. Emotional function, however, was not scored different from NN (data not shown). As NI children have more (co-)morbidities and associated disabilities they will likely score lower in physical and social functioning. The scores in the domain school functioning may be influenced by the possibility for caregivers or patients to leave these questions open if they are not applicable. If at least 50% is filled out in a specific domain, the score over that domain can, however, still be calculated. In the domain school functioning questions regarding attitude and performance at school were generally not filled out, whereas questions regarding presence/absence due to sickness or hospital visits were almost completely filled out by the caregivers.

Before LARS, children reported a significantly higher HRQoL than their parental proxies. This difference remained when only the child’s self-report was compared to the score of their parents and the scores of the parents with children aged <5 or NI children were not taken into account. After LARS, this difference resolved as parents scored the HRQoL higher than children. It is not entirely clear why this difference in HRQoL resolves after LAR. We hypothesize that before LARS caregivers experience more burden from GERD on their child’s HRQoL compared to their children’s own perception. After LARS, GERD resolves in almost all children and it may therefore be possible that HRQoL assessments after LARS are therefore comparable.

Various studies on HRQoL in children indicate that information provided by caregivers does not always correspond to what children report themselves [33, 34]. Pediatric patient self-report is considered to be the standard for measuring HRQoL, as it is the only genuine patient-reported outcome [35]. It can, however, be difficult to obtain self-reports in young children and children with impaired neurodevelopment. In these cases, a parental proxy report may be the only way to assess HRQoL [36]. Furthermore, it has been shown that the parents’ perception of their child’s HRQoL influences health care utilization more than the perception of the child itself [37, 38].

Age at the time of operation was a statistically significant predictor of improvement in HRQoL after LARS. This means that LARS has more effect on HRQoL in older children and may suggest caution when younger children are referred for therapy-resistant GERD. It has been suggested that recurrence of GERD and even the necessity for redo-fundoplication are more frequently seen in young patients. These suggestions were based on two retrospective studies both using regression analysis to identify risk factors [39, 40]. Bearg et al. [39] showed that redo-fundoplication is significantly more frequent if patients are younger or have retching. Ngerncham et al. [40] reported that age less than 6 years was independently associated with increased risk of recurrence of GERD. Furthermore, it may also be possible that older children can specify their (reflux) complaints better, allowing a more precise diagnosis of therapy-resistant GERD to be made. Finally, it has been hypothesized that a young child may outgrow its fundoplication [4].

We initially hypothesized that preoperative gastric emptying might influence the success of LARS and thereby the effect on HRQoL as this has been shown in adult literature [41]. In this study, however, we did not find an effect of gastric emptying on HRQoL.

It is plausible that reflux symptoms and acid exposure influence HRQoL assessment. In the current study, reflux symptoms were negatively associated with lower HRQoL. Remarkably, however, recurrence or persisting pathological acid exposure did not significantly influence HRQoL. It has been described before that reflux symptoms do not correlate to objective measurements of GERD, which may underscore the difficulty in symptom assessment [3, 4, 42].

One of the limitations in the current study was the limited number of 25 patients included. It was therefore only possible to investigate 5 determinants in linear regression assuming that we have sufficient statistical power with 5 patients per predictor. If more patients were included in this study, we would have had more power to detect the influence of these variables, and we had been able to investigate more potential determinants of changes in HRQoL. Secondly, this limited sample size, in addition to variations in surgical technique and multiple institutions, results to various forms of potential bias, such as confounding or type 2 errors. Furthermore, not all patients were able to fill out the self-report because of impaired neurodevelopment or age and as mentioned previously not all questions could be filled out by their caregivers, because in specific domains the questions were not always suitable when considering comorbidities and patient’s limitations.

In conclusion, health-related quality of life, scored by both pediatric patients and their caregivers, significantly improves after LARS. Although patients with impaired neurodevelopment have lower overall HRQoL compared to neurologically normal developed patients, neurodevelopment itself is not a predictor of inferior improvement in HRQoL after LARS. Older children have a more favorable outcome of LARS on HRQoL compared to younger children. This suggests that with the diagnosis of therapy-resistant GERD in younger children, one should possibly be cautious to perform LARS.
